# Season, household registry and isolated birth defects: a population-based case-control study in Danyang, China

**DOI:** 10.1093/inthealth/ihae034

**Published:** 2024-05-27

**Authors:** Shuhan Miao, Liqun Liu, Yanlin Tang, Hongyan Ge

**Affiliations:** D epar tment of Health Care, Women and Children Health Hospital of Zhenjiang, No. 20, Zhengdong Road, Zhenjiang 212003, China; Department of Preventive Medicine and Public Health Laboratory Science, School of Medicine, Jiangsu University, No. 301, Xuefu Road, Zhenjiang 212013, China; Department of Preventive Medicine and Public Health Laboratory Science, School of Medicine, Jiangsu University, No. 301, Xuefu Road, Zhenjiang 212013, China; D epar tment of Health Care, Women and Children Health Hospital of Zhenjiang, No. 20, Zhengdong Road, Zhenjiang 212003, China

**Keywords:** birth defects, epidemiology, risk factors

## Abstract

**Background:**

A birth population-based study was conducted in Danyang, Jiangsu Province, to evaluate major birth defects in emerging regions in China with similar maternal and neonatal care conditions.

**Methods:**

We conducted a population-based study in a cohort of infants born in Danyang from 2014 to 2021, including 55 709 perinatal infants. Four categories of isolated birth defects were defined as cases: congenital heart defects (CHDs; n=2138), polydactyly (n=145), cleft lip with or without palate (CL/P; n=76) and accessory auricles (n=93). Infants with congenital malformations were identified by the Chinese Birth Defects Monitoring Network.

**Results:**

Compared with autumn, conception in spring (OR=1.31 [1.16–1.48]) and winter (OR=1.39 [1.23–1.58]) was associated with an increased risk of CHD. Increased risk of CHD, CL/P and accessory auricles was significantly associated with non-local registered residence (OR=1.17 [1.07–1.28], OR=2.73 [1.52–4.88] and OR=2.11 [1.20–3.71], respectively). Individuals of Han nationality were less likely to have polydactyly (OR=0.23 [0.05–0.98]).

**Conclusions:**

The season of pregnancy was significantly associated with CHDs. Offspring of mothers with non-local registered hometown had greater risks of CHDs, CL/P and accessory auricles.

## Introduction

Birth defects (BDs) are the major source of early miscarriage, perinatal death, infant mortality and child disability, and constitute an important global public health issue. BDs are defined as functional, structural and metabolic diseases^1–4^ that are established during or before birth. Multiple defects may be found in several organs of children with BDs.^5–7^ The prevalence of BDs has been reported to vary widely between different geographic locations, with reported rates of 3.13 per 100 in the USA, 1.50 per 100 in Japan, 2.39 per 100 in Europe and 2.07 per 100 in Turkey.^2,8–11^ Studies have shown that BDs are associated with maternal age, birth weight, the mother's education level, pregnancy time, alcohol consumption and maternal race.^1,2,6^ Although behavioral and environmental components play an important role in the etiology of BDs, substantial ambiguity persists regarding this subject.

Danyang has a population of 828 316, and it is the largest functional area in Zhenjiang City, Jiangsu Province, China. Danyang contains 221 200 women aged 15–49 y, including 162 160 married women. Given that etiological studies on congenital defects in eastern China have rarely been reported in recent years, the current study sought to fill an important knowledge gap by focusing on Danyang, and to evaluate the types and risk factors of BDs in emerging regions in China with similar maternal and neonatal care conditions.

## Materials and Methods

### Study population

This study was a population-based case-control study in a cohort of infants born in Danyang district from 1 January 2014 to 31 December 2021. All included cases with BDs presented from 28 wk gestation to 42 d after birth, and fetus malformations that presented before 28 wk gestation were also recorded in the surveillance system. Infants with congenital malformations were identified by the Chinese Birth Defects Monitoring Network. Procedures for data collection, data filling and quality controls have been reported in detail by Dai et al.^12^

### Surveillance data

Surveillance data of BDs were collected from all hospitals with obstetrics departments, neonatal departments or pediatric departments. All infants and fetuses were examined carefully by trained professionals using routine obstetric diagnosis, physical examination or autopsies. The surveillance data were collected by professional obstetrics and gynecology doctors, pediatricians or neonatal doctors from these hospitals. Each card recorded the maternal information (including maternal age, gravidity, parity, ethnicity, household registry), neonatal birth information (including infant gender, birth weight, birth season, fetus number, birth outcome) and diagnosis of BDs. Case report cards, which were reported both on paper and online, were reviewed and audited by maternal/child health hospitals and health administrative departments, respectively. Periodical quality controls of the monitored hospitals were inspected and examined once every quarter at the county level and half-yearly at the city level or province level to reduce misstatement or failure to report.

### Inclusion and exclusion criteria

The diagnosis of BDs was based on the Chinese National Criteria of Birth Defects and Tiny Deformities stated in the Manual.^13^ BDs were classified into different groups based on the International Classification of Diseases, 10th Revision (ICD-10), including those for fetal nervous system malformation, congenital malformations of the eye, ear and neck, circulatory system congenital malformation, digestive system congenital malformation, urinary system malformation, musculoskeletal system congenital malformation, chromosomal abnormalities and other defects.^7^ In the current study, cases were defined as perinatal infants with confirmed isolated congenital heart defects (CHDs), polydactyly, cleft lip with or without palate (CL/P) or accessory auricles. These represented the top four phenotypes of BDs in Danyang district. The control group was composed of all non-malformed perinatal infants born in the area from which the cases were recruited during the same period.

Maternal age (<35, ≥35 y), gestational age (<32, 32–37, ≥38 wk), plurality (single, twins), ethnicity (Han, other), maternal gravidity (1, 2, ≥3 times), birth weight (<2500, 2500–3999, ≥4000 g), birth season (spring [March to May], summer [June to August], autumn [September to November] and winter [December to February]), household registry (local registered residence for those who achieved permanent residency, non-local registered residence for those who lived in Danyang for >1 y without a permanent residence permit) and perinatal outcomes (live birth, 0–42 days death, fetal death and stillbirth) were obtained for all births and BDs.

### Statistical analysis

All statistical analyses were conducted using SPSS, version 22.0 (Chicago, IL, USA). χ^2^ tests were used for showing the difference between isolated BDs (CHD, polydactyly, CL/P or accessory auricles) and controls. We used multiple logistic regression analysis to show the associations between possible risk factors and BDs, which included adjustments for covariates that were regarded as plausible confounders (covariates that were differentially distributed between case group and control group). Household registry, birth season and nationality were entered into a simultaneous model along with the other listed covariates. Linear regression analysis using a stepwise procedure was carried out to test collinearity (gestational age and birth weight) prior to multivariable logistic analysis. Variance inflation factor (VIF)≥10 was indicated as strong collinearity. If so, only one significant covariate was selected in our multiple logistic regression analysis. p<0.05 was considered to indicate statistical significance.

## Results

### Demographic characteristics

From 1 January 2014 to 31 December 2021, a total of 55 709 deliveries were reported to the Birth Defects Monitoring Network of the Danyang Prefecture of Zhenjiang City. We examined the prevalence rates of the four most common BDs.

Our analysis included 2138 cases of CHDs, 145 cases of polydactyly, 76 cases ofion CL/P and 93 cases of accessory auricles. Table 1 provides a summary of the selected maternal variables. The results revealed that mothers of infants with CHDs generally had a higher maternal age compared with the control group (10.38% vs 7.96%, p<0.01). Additionally, the frequency of non-Han nationality mothers was significantly higher in the polydactyly group (2.76%) compared with that in the control group (0.83%, p<0.05). A higher percentage of maternal gravidity ≥3 was observed in CHD-affected mothers compared with control-mothers (36.62% vs 32.31%, p<0.01). Moreover, a higher percentage of CHD (33.16%), CL/P (56.58%) and accessory auricles (43.01%) case-mothers were non-residents compared with the proportion of control-mothers who were non-residents (29.76%, p<0.01).

**Table 1.** Distribut of significant maternal variables between birth defects cases and controls

	CHD	Polydactyly	CL/P	Accessory auricles	Control
Characteristics	N (%)	N (%)	N (%)	N (%)	N (%)
Maternal age (y)	**				
<35	1916 (89.62)	129 (88.97)	69 (90.79)	84 (90.32)	49 014 (92.03)
≥35	222 (10.38)	16 (11.03)	7 (9.21)	9 (9.68)	4243 (7.96)
Nationality		*			
Han	2113 (98.83)	142 (97.24)	75 (98.68)	91 (97.85)	52 817 (99.17)
Other	25 (1.17)	3 (2.76)	1 (1.32)	2 (2.15)	440 (0.83)
Maternal gravidity	**				
1	830 (38.82)	60 (41.38)	31 (40.79)	30 (32.26)	21 668 (40.69)
2	525 (24.56)	34 (23.45)	16 (21.05)	26 (27.96)	14 381 (27.00)
≥3	783 (36.62)	51 (35.17)	29 (38.16)	37 (39.78)	17 208 (32.31)
Household registry	**		**	**	
Local registered residence	1429 (66.84)	99 (68.28)	33 (43.42)	53 (56.99)	37 407 (70.24)
Non-resident residence	709 (33.16)	46 (31.72)	43 (56.58)	40 (43.01)	15 850 (29.76)

Abbreviations: CHD, congenital heart defect; CL/P, cleft lip and palate.

*p<0.05; **p<0.01 compared with controls.

As shown in Table 2, infants with CHDs and CL/P were more likely to give birth prematurely and had a lower birth weight than those without malformation (p<0.01). A predominance of males was observed in the CL/P (59.21%) and polydactyly (62.07%) groups compared with the proportion of males in the control group (52.03%, p<0.05). The risk of CHDs (4.02%) and accessory auricles (7.53%) was higher in the multiple pregnancy groups compared with controls (2.28%, p<0.01). The frequency of live births was also statistically lower in the CHD (96.82%) and CL/P (63.16%) groups compared with that in the control group (99.83%, p<0.01).

**Table 2.** Distribution of significant newborn demographic between birth defects cases and controls

	CHD	Polydactyly	CL/P	Accessory auricles	Control
Characteristics	N (%)	N (%)	N (%)	N (%)	N (%)
Gestational age (wk)	**		**		
<32	79 (3.70)	1 (0.69)	26 (34.21)	0 (0.00)	208 (0.39)
32–37	405 (18.94)	22 (15.17)	6 (7.90)	14 (15.05)	5882 (11.04)
≥38	1654 (77.36)	122 (84.14)	44 (57.89)	79 (84.95)	47 167 (88.56)
Plurality	**			**	
Singleton	2052 (95.98)	143 (98.62)	73 (96.05)	86 (92.47)	52 045 (97.72)
Multiple	86 (4.02)	2 (1.38)	3 (3.95)	7 (7.53)	1212 (2.28)
Birth season	**				
Spring	588 (27.50)	33 (22.76)	18 (23.68)	16 (17.20)	13 178 (24.74)
Summer	549 (25.68)	43 (29.66)	16 (21.05)	26 (27.96)	15 218 (28.57)
Autumn	462 (21.61)	32 (22.07)	17 (22.37)	25 (26.88)	13 536 (25.42)
Winter	539 (25.21)	37 (25.52)	25 (32.89)	26 (27.96)	11 325 (21.26)
Gender		*	*		
Male	1101 (51.50)	90 (62.07)	45 (59.21)	48 (51.61)	27 708 (52.03)
Female	1037 (48.50)	55 (37.93)	31 (40.79)	45 (48.39)	25 549 (47.97)
Birth weight (g)	**		**		
<2500	233 (10.90)	7 (4.83)	30 (39.47)	2 (2.15)	2008 (3.77)
2500–3999	1646 (76.99)	129 (88.97)	40 (52.63)	86 (92.47)	46 259 (86.86)
≥4000	259 (12.11)	9 (6.21)	6 (7.89)	5 (5.38)	4990 (9.37)
Outcome	**		**		
Live birth	2070 (96.82)	143 (98.62)	48 (63.16)	93 (100.00)	53 166 (99.83)
Fetal death and stillbirth	54 (2.53)	2 (1.38)	25 (32.89)	0 (0.00)	53 (0.10)
0–42 days death	14 (0.65)	0 (0.00)	3 (3.95)	0 (0.00)	37 (0.07)

Abbreviations: CHD, congenital heart defect; CL/P, cleft lip and palate.

*p<0.05; **p<0.01 compared with controls.

Poor outcome of pregnancy contained fetal death and stillbirth and 0–42 days death.

Because the linear regression results had excluded the possibility of strong co-linearity (VIF<10) between gestational age and birth weight, the above-mentioned and other significantly incomparable covariates were considered as biologically potential confounders that were entered into the adjusted model.

### Association between risk factors and BDs

As shown in Table 3, the risk of CHDs (OR=1.17 [1.07–1.28]), CL/P (OR=2.73 [1.52–4.88]) and accessory auricles (OR=2.11 [1.20–3.71]) was much higher among mothers with non-resident residence status compared with that in mothers with local registered residence status. Compared with conceptions in autumn, conceptions in spring (OR=1.31 [1.16–1.48]) and winter (OR=1.39 [1.23–1.58]) were associated with a higher risk of CHDs. Infants of Han nationality were less likely to have polydactyly compared with those of other nationalities (OR=0.23 [0.05–0.98]).

**Table 3.** Basic characteristics of cases with isolated birth defects and unaffected controls

	CHD		CL/P		Polydactyly		Accessory auricles	
Characteristics	OR	95% OR	OR	95% OR	OR	95% OR	OR	95% OR
Season	**							
Spring	1.31	1.16–1.48	0.75	0.39–1.46	1.23	0.70–2.19	1.29	0.62–2.68
Summer	1.06	0.93–1.20	0.66	0.33–1.33	0.86	0.46–1.61	0.84	0.38–1.88
Autumn	–	–	–	–	–	–	–	–
Winter	1.39	1.23–1.58	0.52	0.25–1.10	0.75	0.39–1.46	0.95	0.44–2.05
Household registry	*		**				**	
Non-resident residence	1.17	1.07–1.28	2.73	1.52–4.88	1.25	0.74–2.13	2.11	1.20–3.71
Local registered residence	–	–	–	–	–	–	–	–
Nationality					*			
Han	0.70	0.47–1.06	0.63	0.08–5.16	0.23	0.05–0.98	0.49	0.07–3.66
Other	–	–	–	–	–	–	–	–

Abbreviations: CHD, congenital heart defect; CL/P, cleft lip and palate.

*p<0.05; **p<0.01 compared with controls.

Adjusted for maternal age, maternal gravidity, gestational age, plurality, gender, birth weight, outcome, season, household registry and nationality.

## Discussion

BDs were identified as a major cause of infant mortality, resulting in 1.2 deaths per 1000 live births.^14^ Among BDs, CHDs were found to be the most prevalent, in accord with the current findings.^7,15^

The current study revealed that the prevalence of BDs was higher in high-risk groups of pregnant women and children, such as those with premature births, low birth weight, multiple births and poor pregnancy outcomes. This finding is consistent with previous research conducted by Kim et al. in Korea.^16^ A study conducted in Guangdong, China, also indicated that the prevalence of CHDs increased with maternal age.^17^ Furthermore, Tanner et al. reported that the risk of CHD was 2.4 times higher in premature infants compared with that in mature infants. Other research has shown a reciprocal relationship between the presence of BDs and being born preterm.^2,18^ However, surprisingly, most infants in the current study had normal birth weight, in contrast to many previous studies reporting a higher risk of BDs with low birth weight.^16,19^ Multiple pregnancy has been widely reported to be associated with an increased risk of many BDs, including hydrocephalus, anencephaly, CL/P, CHD, anorectal atresia and hypospadias.^20–23^ Interestingly, the current study indicated that multiple pregnancy increased the risk of CHDs and accessory auricles, but had no effect on CL/P or polydactyly. Proposed mechanisms include insufficient nutrition supply, crowding, vascular interchange and immature assisted reproductive technology.^22,24,25^

The average prevalence of CHDs, CL/P and accessory auricles among non-local registered residents was significantly higher than that among local registered residents. In Danyang, the majority of non-locally registered pregnant women are factory workers, primarily in shoe factories and textile mills. Compared with locally registered pregnant women, these non-local residents are at a higher risk of occupational exposure, potentially coming into contact with organic solvents, inhalable particles and other risk factors for BDs in their workplaces.^26^ In addition, non-local residents typically earn lower wages and have lower levels of education.^27^ The higher prevalence of BDs among non-local residents may be attributed to a lack of overall health awareness, limited access to medical services, inadequate nutrition and a lack of access to quality healthcare services.

Seasonal patterns have been found to be correlated with various aspects of health and development, such as body height, birth outcomes, mental health, life expectancy, intelligence quotient, educational attainment and income.^28^ For instance, it has been demonstrated that there are peaks in CHDs during the spring and winter months, particularly among women living in rural areas, suggesting potential exposure to environmental risk factors, such as agricultural chemicals, during these times.^29,30^ Other factors, such as infections, nutrition and high temperatures, may also play a role in the increased frequency of BDs during certain seasons.^31,32^ It has been hypothesized that these seasonal variations may result from the interaction of multiple environmental risk factors. Recent studies have indicated that several types of BDs exhibit seasonal variation, with conditions like microtia peaking in autumn and winter, anencephaly exhibiting a peak in March to August and CL/P exhibiting a significant peak incidence in August and September.^33–36^ However, differences in the seasonal incidence of CL/P, polydactyly and accessory auricles have not been observed. Polydactyly is one of the most common limb malformations in China, yet there is relatively limited epidemiological investigation data available for Chinese populations.^37^ The current findings indicated that Han ethnicity was associated with a reduced risk of polydactyly, providing valuable epidemiological information on this condition. This suggests that further investigation into the seasonal patterns of BDs in rural areas, particularly related to maternal exposure to agricultural chemicals, could be worthwhile.

While we have analyzed certain social population structures and confounding factors, it is important to acknowledge that the potential influence of other unknown factors, such as maternal health status, dietary intake, family history, reproductive history and environmental risk exposure during pregnancy, requires further investigation. As a result, there is a possibility that some of the risk estimates in our study were overestimated. This could have contributed to the significant risk estimates reflected in the wide confidence limits observed in our study.

In conclusion, the current findings suggest that gestational age <32 wk, birth weight <2500 g, maternal age ≥35 y, multiple pregnancies, non-Han nationality and poor pregnancy outcomes are associated with an increased incidence of BDs. Additionally, our findings suggest that the season of conception, household registry and ethnicity may also play a significant role in the development of certain BDs. By examining the prevalence of these defects in Danyang, our research may support the development of more comprehensive strategies by other researchers to reduce the occurrence of BDs in the future.

## Data Availability

The authors confirm that the data supporting the findings of this study are available within the article.
